# Preclinical study of human umbilical cord mesenchymal stem cell sheets for the recovery of ischemic heart tissue

**DOI:** 10.1186/s13287-022-02919-8

**Published:** 2022-06-11

**Authors:** Shuang Gao, Yongqiang Jin, Jianlin Ma, Juan Wang, Jing Wang, Zehua Shao, Taibing Fan, Mingkui Zhang, Dehua Chang

**Affiliations:** 1 BOE Regenerative Medicine Technology Co., Ltd., No. 9 JiuXianQiao North Road, Beijing, 100015 China; 2 Heart Center, First Hospital of Tsinghua University, No. 6 JiuXianQiao 1st Road, Beijing, 10016 China; 3 Heart Center of Henan Provincial People’s Hospital, Zhengzhou University People’s Hospital, No. 7 Weiwu Road, Zhengzhou, 450003 China; 4 Children Heart Center, Fuwai Central China Cardiovascular Hospital, No. 1 Fuwai Road, Zhengzhou, 450018 China; 5 Department of Cell Therapy in Regenerative Medicine, The University of Tokyo Hospital, 7-3-1 Honggo, Bunkyo-ku, Tokyo, 113-8655 Japan

**Keywords:** Umbilical cord mesenchymal stem cells, Cell sheet, Myocardial infarction, Ischemic myocardial tissue, Safety of cell sheet

## Abstract

**Background:**

Human umbilical cord mesenchymal stem cells (hUC-MSCs) have been widely used due to their multipotency, a broad range of sources, painless collection, and compliance with standard amplification. Cell sheet technology is a tissue engineering methodology requiring scaffolds free, and it provides an effective method for cell transplantation. To improve the therapeutic efficacy, we combined hUC-MSCs with cell sheet technology to evaluate the safety and efficacy of hUC-MSC sheets in preclinical studies using appropriate animal models.

**Methods:**

hUC-MSC sheets were fabricated by hUC-MSCs from a cell bank established by a standard operation process and quality control. Cytokine secretion, immunoregulation, and angiopoiesis were evaluated in vitro. Oncogenicity and cell diffusion assays of hUC-MSC sheets were conducted to verify the safety of hUC-MSCs sheet transplantation in mice. To provide more meaningful animal experimental data for clinical trials, porcine myocardial infarction (MI) models were established by constriction of the left circumflex, and hUC-MSC sheets were transplanted onto the ischemic area of the heart tissue. Cardiac function was evaluated and compared between the experimental and MI groups.

**Results:**

The in vitro results showed that hUC-MSC sheets could secrete multiple cellular factors, including VEGF, HGF, IL-6, and IL-8. Peripheral blood mononuclear cells had a lower proliferation rate and lower TNF-α secretion when co-cultured with hUC-MSC sheets. TH1 cells had a smaller proportion after activation. In vivo safety results showed that the hUC-MSCs sheet had no oncogenicity and was mainly distributed on the surface of the ischemic myocardial tissue. Echocardiography showed that hUC-MSC sheets effectively improved the left ventricular ejection fraction (LVEF), and the LVEF significantly changed (42.25 ± 1.23% vs. 66.9 ± 1.10%) in the hUC-MSC transplantation group compared with the MI group (42.52 ± 0.65% vs. 39.55 ± 1.97%) at 9 weeks. The infarct ratio of the hUC-MSCs sheet transplantation groups was also significantly reduced (14.2 ± 4.53% vs. 4.00 ± 2.00%) compared with that of the MI group.

**Conclusion:**

Allogeneic source and cell bank established by the standard operation process and quality control make hUC-MSCs sheet possible to treat MI by off-the-shelf drug with universal quality instead of individualized medical technology.

**Supplementary Information:**

The online version contains supplementary material available at 10.1186/s13287-022-02919-8.

## Introduction

Ischemic heart disease is one of the leading causes of morbidity and mortality worldwide. It is characterized by an imbalance between myocardial oxygen supply and demand, eventually leading to fatal heart failure (HF) and death of 30–40% patients in East Asia and more than 50% in Europe and North America [1]. Over the past half a century, conventional medicine and cardiac surgery have offered great breakthroughs and progress, resulting in a dramatic decline in HF mortality [2]. However, even with the major advances, medical or cardiac surgical treatment of HF only temporarily delays the progressive disease process, with the only definite cure being an artificial heart or heart transplantation. Transplantation of artificial hearts is accompanied by high medical costs and postoperative bleeding, infection, and other complications. Although allogeneic heart transplantation provides hope of recovery for end-stage HF patients, a shortage of sources, endemic infections, and high medical expenses limit its application on a large scale [3].

The idea of using mesenchymal stem cells (MSCs) has emerged in the last decade as a leading approach for a regenerative medicine strategy to address cardiovascular diseases such as HF. MSCs are multipotent cells that are easily isolated, expanded, and immunologically tolerated, allowing them to be allogeneic. As a “live” drug, transplanted MSCs could continuously work through many mechanisms, including paracrine modulation, cell migration, and trans-differentiation. Many previous studies have applied MSCs to ischemic or non-ischemic heart disease, and their safety and efficacy have been verified in both preclinical and early phase clinical trials [4–7].

MSC suspensions are a common application route for their convenient delivery, such as intravenous infusion, percutaneous intracoronary infusion, and peripheral intravenous infusion [8]. However, after infusion of a suspension, generally less than 10–20% of the injected cells are found at the injured area within a few hours or days after delivery, and only a few cells actively engraft into the affected tissue. Suspension cell injection causes significant loss and death of cells and uneven local distribution, and the low survival of transplanted cells reduces their therapeutic effects [9].

Cell sheet technology eliminates the problem of retention, helps retain the transplanted cells at the desired location, and provides an appropriate lifespan for transplanted MSCs [10]. The technology was developed by Prof. Okano’s team using thermo-responsive culture dishes coated with poly(N-isopropylacrylamide). With this application, cells adhere to and proliferate on the culture dish surface at 37 °C, and a cell sheet with extracellular matrix (ECM) detaches at temperatures lower than 32 °C [11, 12]. Using this technology, MSC sheets were prepared and transplanted into porcine [13, 14] and rodent MI models [10, 15–18]. The results showed that MSC sheets effectively improved left ventricular (LV) function and attenuated LV remodelling.

MSCs most commonly used in clinical studies to date originate from bone marrow, adipose tissue, and umbilical cord, among other sources [19]. However, after decades of basic and clinical research, the overall benefit and the best cell source remain unresolved. Most basic and clinical studies have used bone marrow mesenchymal stem cells (BM-MSCs) or adipose tissue mesenchymal stem cells (AD-MSCs); nonetheless, these cells present disadvantages for clinical applications, such as an invasive harvesting procedure, decreased proliferation, and a differentiation potential related to donor age and comorbidities [20]. Umbilical cord mesenchymal stem cells (UC-MSCs) were isolated from the connective tissue of the umbilical cord in the early 1990s and are an ideal cell source of MSCs for stem cell transplantation [21]. UC-MSCs are easily attainable and expanded in vitro, and their collection is devoid of ethical concerns [22]. UC-MSCs have undergone barely any cellular ageing, and their proliferative capacity and cell viability are better than those of BM-MSCs and AD-MSCs, even under hypoxic conditions [22]. Bartolucciet et al. also demonstrated that UC-MSCs possess a superior migration capacity and higher hepatocyte growth factor (HGF) secretion than BM-MSCs [4]. Moreover, MHC II antigens correlated with alloimmune rejection were barely expressed by UC-MSCs compared to other comparator BM-MSCs and AD-MSCs [23]. Since human UC-MSCs (hUC-MSCs) can be frozen and stored long-term, it is possible to establish a cell bank of hUC-MSCs according to uniform sample selection and a standard operating procedure [24]. The cell bank could also be managed with a unified quality standard, to provide safe and high-quality hUC-MSCs for cell sheet manufacture.

For the management of cell banks, safety and efficiency evaluations are two preconditions for the clinical application of hUC-MSCs sheet. Our previous study demonstrated the effectiveness of hUC-MSCs sheet in cardiac function recovery in an MI mouse model [25].

In this study, an hUC-MSC bank was established according to a uniform quarantine standard and culture process. hUC-MSC sheets were fabricated, and their structure, cytokine secretion, immune regulation, and angiogenesis were tested in vitro. Tumorigenicity and cell diffusion after hUC-MSCs sheet transplantation were evaluated to confirm the safety of the hUC-MSCs sheet. Furthermore, the hUC-MSC sheet was transplanted into the MI porcine model to evaluate its effectiveness in improving cardiac function.

## Materials and methods

### Isolation and characterization of hUC-MSCs

Umbilical cord samples were collected from donors during full-term childbirth. Before parturition, puerperas tested negative for HIV, HBV, HCV, HCMV, EBV, HTLV, HPV, and human parvovirus B19. Umbilical cord samples were preserved in sterile saline at 4 °C after collection and transferred to the laboratory within 24 h.

For hUC-MSC primary culture, the explant method was used as described by Lei et al. [26]. Briefly, veins, arteries, and tunica externa of the umbilical cord were removed to obtain Wharton’s jelly. Next, Wharton’s jelly was minced into small fragments and uniformly placed in culture dishes (Φ100 mm, Corning, Manassas, USA). Finally, culture medium (α-MEM medium (Corning) with 10% (v/v) FBS (Life, NY, USA) and 40 unit/ml gentamicin) was added to the culture dishes. The culture dishes were placed in an incubator at 37 °C, 5% (v/v) CO_2_, and 95% humidity. The culture medium was replaced every 3 days until the cells migrated from the tissue fragments and reached 70% confluence. The tissues were removed, and the cells were digested by TrypLE (Life) and collected for further culture after diluting at a 1:4 ratio.

hUC-MSCs were passaged when cell confluence reached 70–80%. The 1st and 4th passage cells were preserved in liquid nitrogen circumstances and called the main cell bank and working cell bank, respectively.

### Identification of hUC-MSCs

During the passaging of hUC-MSCs, their morphology was observed by an inverted microscope (CKX41, OLYMPUS, Tokyo, Japan).

To test the multilineage differentiation capacity of the hUC-MSCs, adipogenic, chondrogenic, and osteogenic differentiation kits (VivaCell, Shanghai, China) were used following the manufacturer’s specifications. Finally, adipogenic, chondrogenic, and osteogenic differentiation results were tested through Oil red O staining, Alcian blue staining, and Alizarin red staining, respectively.

Surface markers on hUC-MSCs were evaluated by flow cytometry (Canto II, BD Biosciences, CA, USA). Briefly, hUC-MSCs were washed twice with staining buffer (BD Biosciences) and stained with anti-CD11b-PE, anti-CD19-FITC, anti-CD34-PE, anti-CD45-APC-Cy7, anti-CD73-PE, anti-CD90-FITC, anti-CD105-APC, anti-HLA-DR-APC, and the corresponding isotype control antibodies (all antibodies were purchased from BD Biosciences) for 30 min at room temperature in the dark. Then, the hUC-MSCs were washed twice with staining buffer and resuspended in staining buffer for flow cytometry analysis.

### Fabrication of hUC-MSCs sheet

**Fig. 1:**
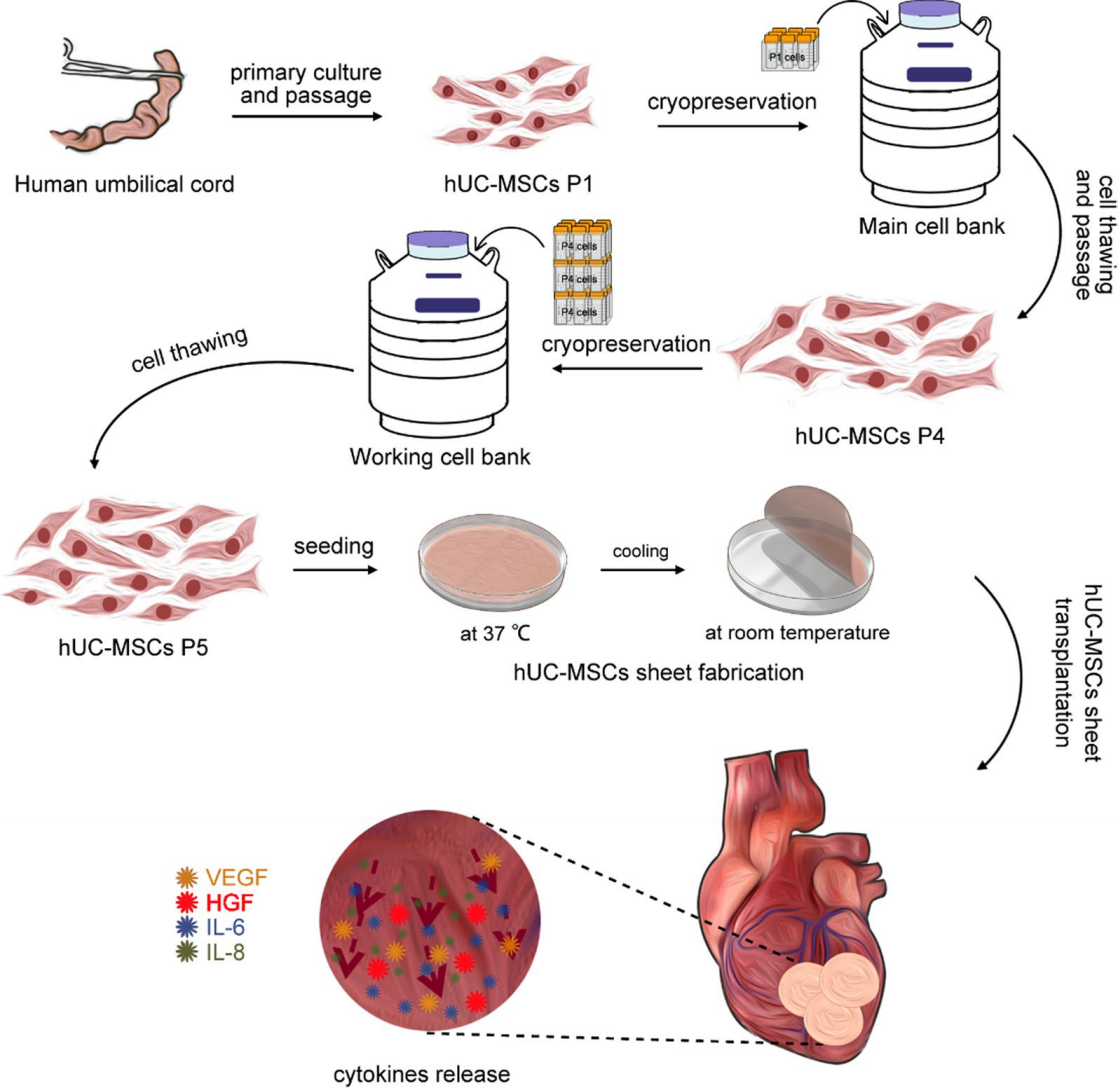
Schematic illustrations of the procedures used for the fabrication of hUC-MSCs sheet and the cytokines secretion from hUC-MSCs sheet to ischemia heart tissue

As shown in the flowchart (Fig. 1), 5th passage hUC-MSCs were obtained after thawing cells stored in the working cell bank. When cell confluence reached approximately 80%, the hUC-MSCs were collected from the flasks and rinsed with PBS. Then, 6 × 10^7^ hUC-MSCs were suspended in the Cell Sheet Culture Medium (BOE Regenerative Medicine Co. Ltd., Beijing, China), seeded onto a Φ100 mm temperature-responsive cell culture dish (Thermo Fisher Scientific, Waltham, MA, USA), and incubated at 37 °C, 5% (v/v) CO_2_, and 95% humidity overnight. After that, the culture dish was moved to a biosafety cabinet at room temperature for 40 min. The hUC-MSCs sheet detached from the culture dish spontaneously.

### Structure characterization of hUC-MSCs sheet

The micro-architectures of the hUC-MSC sheets were analysed by scanning electron microscopy (SEM, Hitachi S-4800, Tokyo, Japan). The hUC-MSCs sheet was fixed with 2.5% (v/v) glutaraldehyde and dehydrated by graded alcohol. Then, samples were mounted on a two-inch aluminium stage and sputtered with gold. Scan settings of 5 keV and 10 mA were used.

For histological analysis, the hUC-MSCs sheet was fixed with 4% paraformaldehyde. Specimens were then embedded in optimal cutting temperature compound (SAKURA, Tokyo, Japan) and cut into 10-μm-thick sections. Fibronectin and integrin-β1 were stained according to normal immunohistochemistry and labelled with FITC. Cell nucleuses were marked by DAPI. For haematoxylin and eosin (H&E) staining, samples were embedded in paraffin and sliced into 5-μm-thick sections, which were treated according to the conventional method.

### In vitro functional evaluation of hUC-MSCs sheet

To evaluate the function of hUC-MSC sheets in vitro, cytokine secretion, immunoregulation, and angiogenesis assays were conducted.

For cytokine secretion testing, hUC-MSC sheets were attached to culture dishes (Φ100 mm, Corning), and 10 ml fresh culture medium was added. The culture dishes were incubated at 37 °C, 5% (v/v) CO_2_, and 95% humidity for 24 h, and the medium was collected for IL-6 (R&D, Minneapolis, USA), IL-8 (R&D), VEGF (R&D), and HGF (Invitrogen, Camarillo, USA) detection by enzyme-linked immunosorbent assay. Fresh culture medium was tested as the control.

To evaluate the immunoregulatory properties of hUC-MSC cell sheets, hUC-MSCs digested from hUC-MSC sheets were cultured in 6-well plates (Corning) and treated with colchicine to suppress proliferation. After PBS solution rinsing, peripheral blood mononuclear cells (PBMCs) were then added to a 6-well plate for coculture at 37 ºC, 5% (v/v) CO_2_, and 95% humidity. The culture medium was 1640 (Corning) with 10% (v/v) FBS. For the lymphocyte proliferation assay, PBMCs were activated with 10 μg/mL PHA-M and labelled with a BrdU-APC Staining Kit (Invitrogen, Camarillo, USA) according to the manufacturer’s instructions. PBMCs were collected for flow cytometry testing. Meanwhile, the concentration of TNF-α in the culture medium was measured by an ELISA kit (Invitrogen). For the Th1 population regulation assay, PBMCs were activated by an e-Bioscience Cell Stimulation Cocktail (Invitrogen) according to the manufacturer’s instructions. After 24 h of co-culture, the PBMCs were collected and stained with anti-CD3-APC (BD Bioscience), anti-CD8-FITC (BD Bioscience), and anti-IFNγ-PE (BD Bioscience). After that, cytometry testing was used to analyse the Th1 population with IFNγ-positive PBMCs in CD3-positive and CD8-negative populations.

In vitro angiogenesis is another test for hUC-MSC functional evaluation. A 48-well plate was coated with Matrigel (100 μl/well, BD Bioscience) and incubated at 37 °C, 5% (v/v) CO_2_, and 95% humidity for 30 min. After that, human umbilical vein endothelial cells (HUVECs, 1 × 10^4^/well in 100 μl) were seeded on Matrigel, and another 100 μl hUC-MSCs sheet culture supernatant was added to the wells. Culture supernatant (100 μl) during hUC-MSCs sheet fabrication was added to each well as experiment groups after removing cell debris through 1000×*g* centrifugation, and fresh Cell Sheet Culture Medium (100 μl) was used as a negative control. Then, the 48-well plate was incubated for 12 h. There were six parallel wells for each sample, and one image was taken of each well. Image analysis was performed using the “Angiogenesis Analyser” plug-in in ImageJ software. “Number of Junctions” and “Total Length” were used as the evaluation indices as previously reported [27].

### In vivo safety evaluation of hUC-MSCs sheet

The oncogenicity experiment schedule is shown in Figure VA. Female BALB/c nude mice were anaesthetized by intraperitoneal injection of pentobarbital sodium at a dose of 60–80 mg/kg. Then, a small piece of hUC-MSCs sheet (approximately 4 × 10^6^ cells per mouse) was transplanted subcutaneously on the right back of the mouse. The sham operation only cuts the skin with no transplant. The human neuroblastoma cell line SH-SY5Y cell suspension (approximately 4 × 10^6^ cells in 0.2 ml saline per mouse) was injected subcutaneously into the right scapula as the positive control. There were 10 animals in each group. For 139 days of observation, the appearance and size of the transplanted sheets, tumors, and nodules were recorded. After observation, the animals were euthanized, and vital organs (brain, heart, liver, spleen, lung, kidney, lymph gland) and tissue at the transplantation site were observed through histopathology after haematoxylin and eosin (H&E) staining.

Cell diffusion assays after hUC-MSC sheet transplantation (Figure VC) were also conducted to evaluate their safety. The MI model of NPG immunodeficient mice was induced according to a previously reported method [25]. Briefly, mice were anaesthetized and orotracheally intubated. Then, the left coronary artery anterior descending branch (LAD) was ligated via left thoracotomy. After that, a small piece of hUC-MSCs sheet (approximately 1 × 10^6^ cells per mouse) was transplanted onto the infarcted area. The sham operation applied only LAD ligation with no transplant. One day, 1 week, 2 weeks, and 4 weeks after surgery, mice were killed for human DNA detection in blood, spinal cord, brain, lung, heart, liver, spleen, kidney, muscle, femur, stomach, duodenum, jejunum, colon, ovary, and prostate through PCR. DNA was extracted from these organs by DNA Extraction Kit (Magen, Beijing, China) following the manufacturer’s instructions, respectively. Primer sequences are shown in Table 1. The PCR amplification condition was 95 ºC for 5 min; then 40 cycles of 95 ºC for 30 s, 60 ºC for 30 s, and 72 ºC for 30 s. Finally, 60 ºC for 30 s was performed. PCR products were separated in a 1.2% agarose gel and visualized by ethidium bromide staining. There were 3 male mice and 3 female mice in each group for each point in time.

**Table 1:** Primer sequence used in Cell diffusion assays

Primer	Sequence
D18S51-F	TTCTTGAGCCCAGAAGGTTA
D18S51-R	GCTACTATGGACTAATATTAGTTTGG
MUS-ACTB-F	ATCGCCATTTTTGTGCTCTT
MUS-ACTB-R	ATTGAAATGATGGCTTTCGC

### Transplantation of hUC-MSCs sheet to porcine MI model

The experimental timeline is shown in Figure VIA. To induce the MI model, male Bama mini-pigs weighing 12–15 kg (3–4 months old) were pre-anesthetized with Zoletil™ 50 (50 mg/ml, Virbac, France) by intramuscular injection at 10 mg/kg, followed by intubation. Maintenance of anaesthesia was achieved by infusion of isoflurane (0–5%) and oxygen with 80 ml tidal volume and a respiratory rate at 20 times/minute. The left thoracotomy was performed through the fourth intercostal space under anesthesia, and an ameroid constrictor was placed around the proximal portion of the left circumflex. Occlusion and reperfusion of the ameroid constrictor achieved ischemic injury, which resulted in building of mini-pig MI model. Holter electrocardiography (ECG) signals were collected before and after ischemic injury induction. Animals were randomly divided into hUC-MSCs sheet transplantation group (*n* = 3) or MI group (*n* = 4). Two weeks later, hUC-MSCs sheet transplantation was performed through the left fourth intercostal space under anesthesia. The pericardium was opened, and the left ventricular lateral wall was dissected out. The hUC-MSCs sheet was picked up by a sterile intestinal depressor and was placed onto the ischemic area of left ventricular free wall. The hUC-MSCs sheet can be attached to the surface of the heart tissue without sutures because of the abundance existence of ECM. Ten minutes later, 3 ml saline was dripped on the top of the hUC-MSCs sheet to confirm that the cell sheets have been remained its physical attachment. Finally, the pericardium of myocardium was overwrapped by suturing and the chest was closed. Mini-pigs in the MI group underwent only left thoracotomy without hUC-MSCs sheet transplantation. Echocardiography was performed at each check time point, as shown in Figure VIA. Ejection fraction (EF), stroke volume (SV), fraction shortening (FS), end systolic volume (ESV), and end diastolic volume (EDV) were measured using echocardiography. Nine weeks after hUC-MSCs sheet transplantation, the animals were euthanized, and their hearts were separated. The infarct tissue of the heart was stained with 2,3,5-triphenyltetrazolium chloride (TTC) solution (1%, v/v, 37 °C). The heart tissue pieces were scanned directly to analyse the infarct ratio of the left ventricle by ImageJ software. In addition, H&E and masson staining were conducted to observe the histological condition and myocardial fibrosis of the LV, respectively. The fibrosis ratio of infarction area was calculated by ImageJ based on masson staining result.

### Statistical analysis

All numerical data were analysed by GraphPad Prism 5 software (GraphPad Software, La Jolla, US). Means ± standard deviation and 95% confidence limits were calculated for each set of results. One-way ANOVA and a *p* value < 0.05 indicated statistical significance.

## Results

### Identification of hUC-MSCs

**Fig. 2:**
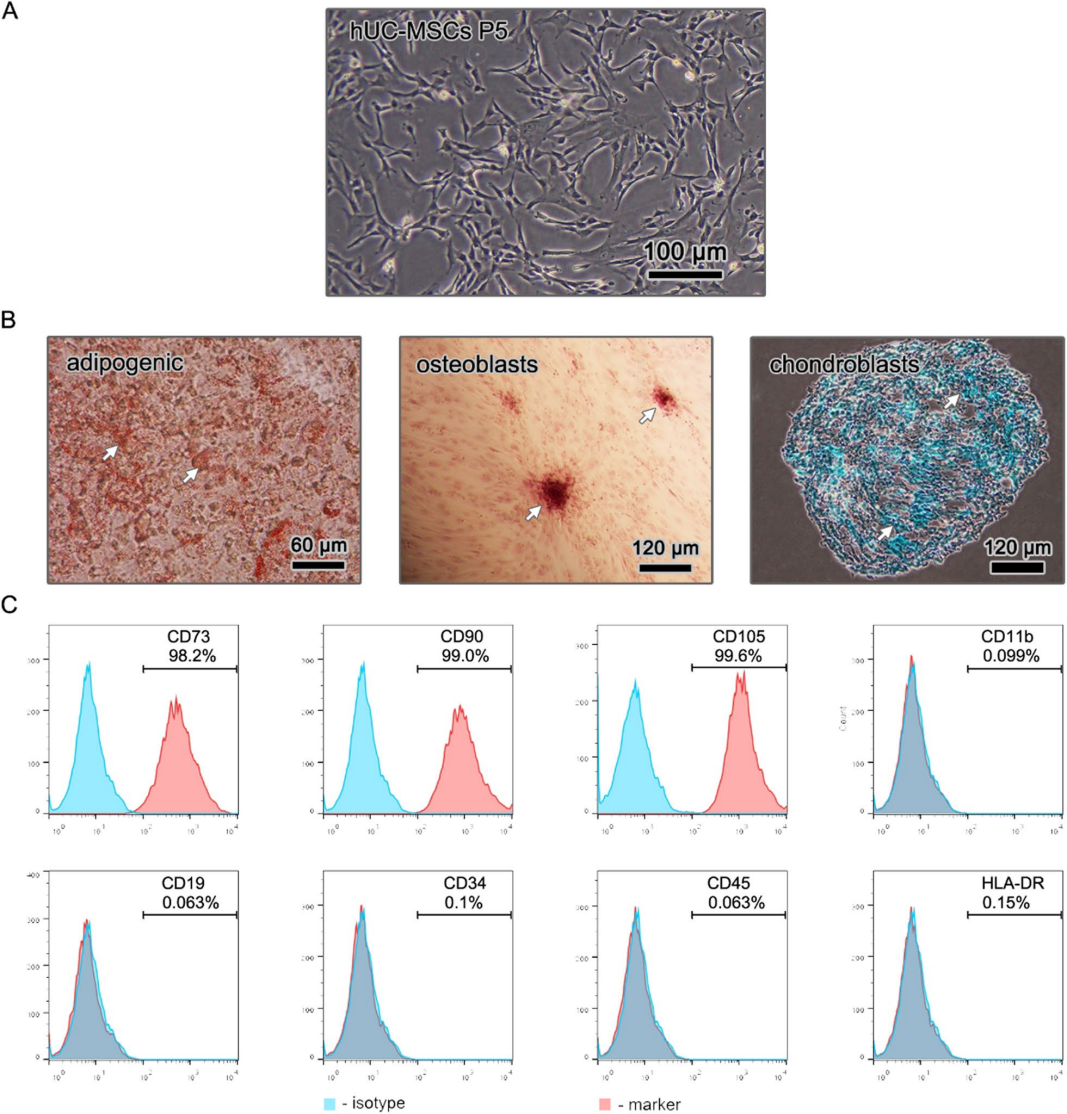
Characterization of isolated hUC-MSCs. **A** Morphology of hUC-MSCs in passage 5 attached on the polystyrene culture dishes; **A** The differentiation capability of hUC-MSCs was confirmed by Oil Red O staining for adipogenesis, Alizarin Red S staining for osteogenesis and Alcian blue staining for chondrogenesis; **C** Immunophenotypic characteristics obtained from flow cytometric analysis indicate that the isolated hUC-MSCs expressed mesenchymal markers such as CD73, CD90 and CD105 but negative for hematopoietic lineage markers CD11b, CD19, CD34, CD45 and HLA-DR

hUC-MSCs were successfully isolated through the explant method and exhibited a spindle type morphology during attached proliferation on a culture flask. Their multi-differentiation potential was also verified. As shown in Fig. 2, hUC-MSCs could be differentiated into adipogenic, chondrogenic, and osteogenic lineages in vitro. The specific staining for each differentiated cell line was positive. Flow cytometry results revealed that hUC-MSCs expressed CD73, CD90, and CD105 positively and CD11b, CD19, CD34, CD45, and HLA-DR negatively. All of these characteristics comply with the criteria defined by the International Society for Cellular Therapy in 2006 [28].

#### Fabrication and characterization of hUC-MSCs sheet

**Fig. 3:**
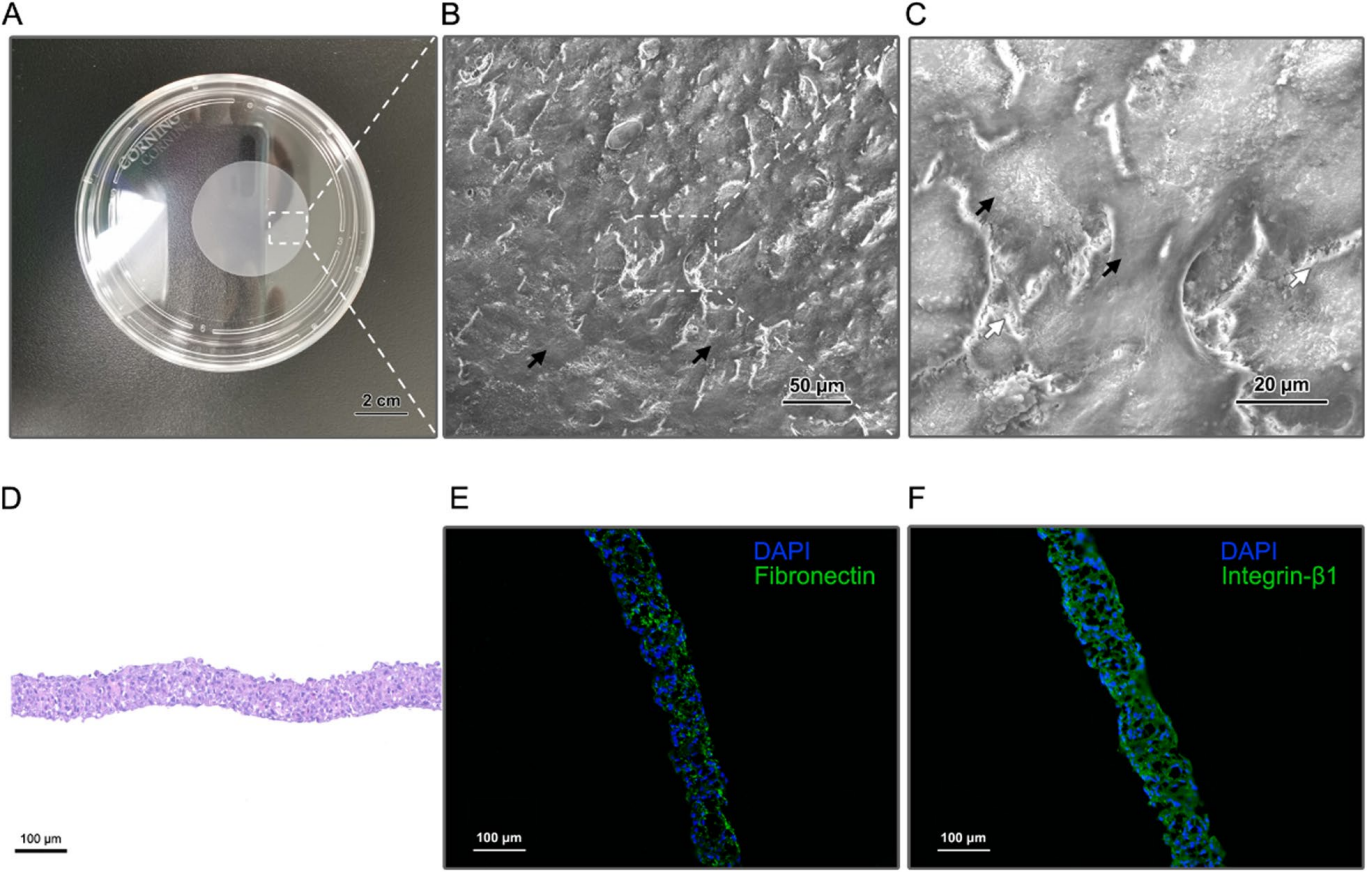
Morphology and structure of the hUC-MSCs sheet. **A** Macro morphology of hUC-MSCs sheet; **B**, **C** Microstructure of hUC-MSCs sheet in different magnifications. Black arrows indicate hUC-MSCs and white arrows indicate ECM junctions between hUC-MSCs; **D** H&E histology of hUC-MSCs sheet in cross-section; Immunofluorescent staining for **E** fibronectin and **F** integrin-β1 of hUC-MSCs sheet

The hUC-MSCs sheet spontaneously detached from the temperature-responsive cell culture dish and then shrink to approximately 4 cm in diameter. The hUC-MSCs sheet exhibited a smooth visual surface (Fig. 3A). SEM observation showed that pavement-like hUC-MSCs (black arrow in Fig. 3B and 3C) were distributed within a well-developed ECM (white arrow in Fig. 3C). A higher-resolution image (Fig. 3C) showed abundant cell–cell and cell-ECM junctions. H&E staining results showed that there were approximately 10 layers of cell stacking in the vertical direction of the hUC-MSCs sheet (Fig. 3D). Here, we also identified the ECM condition and abundance through two representative proteins (fibronectin and integrin-β1) via immunofluorescence staining (Fig. 3E&F).

**Fig. 4:**
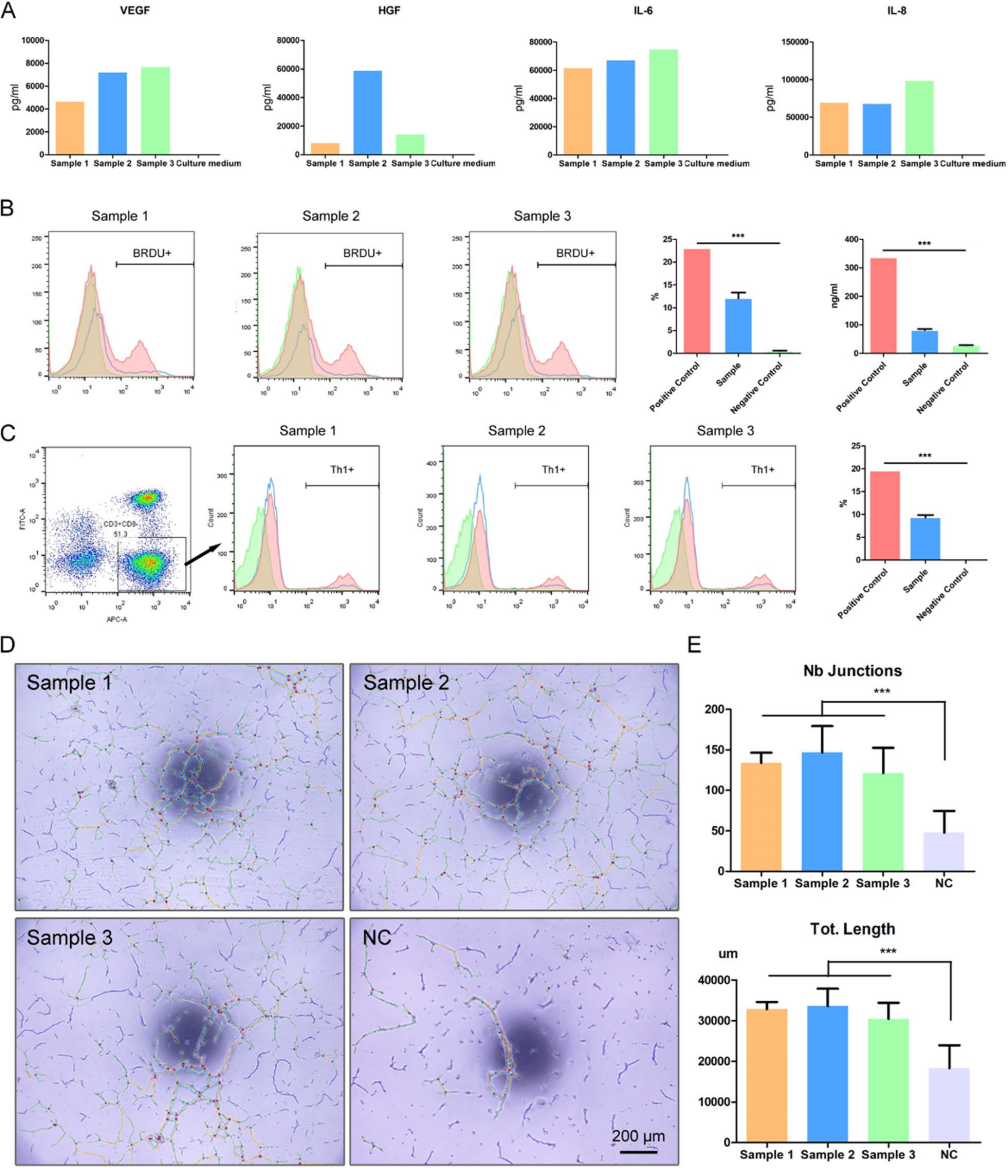
In vitro functional study of hUC-MSCs sheet. **A** Cytokine expression levels of hUC-MSCs sheet; **B** Flow cytometric analyses showed that lymphocytes (labeled by BRDU) had less proliferation rate when co-culturing with hUC-MSCs from hUC-MSCs sheet; **C** lymphocytes co-culturing with hUC-MSCs from hUC-MSCs sheet also had smaller Th1 positive population; **D** Phase contrast images of HUVEC cultured with hUC-MSCs conditional medium and negative control with the superposition of vectorial objects obtained from computer analysis using the customized “Angiogenesis Analyser” for ImageJ; **E** “Number of Junctions” and “Total Length” results calculated by ImageJ. ***p < 0.001

For the function of hUC-MSC sheets, we tested their cytokine secretion (VEGF, HGF, IL-6, IL-8) by ELISA, shown in Fig. 4A. All samples had significant cytokine secretion, while the control culture medium had nearly no such cytokines. However, there existed some variance among the different samples. Sample 2 had much higher HGF secretion than the other two samples. Sample 3 had higher VEGF, IL-6, and IL-8 secretion than the other two samples.

Cytokine secretion was the baseline state; therefore, immunoregulation, and angiogenesis properties of the sheets were tested. Active PBMCs (BRDU positive) had a lower proliferation rate (12.06 ± 1.78%) when co-cultured with hUC-MSC sheets than when cultured alone (23%) (shown in Fig. 4B). In addition, Th1 cells (CD3+, CD8−, INF-γ+) had a smaller proportion in the coculture system (9.27 ± 0.83%) than when cultured alone (19.6%) (shown in Fig. 4C).

For angiogenesis, the hUC-MSCs sheet supernatant had a stronger ability to promote HUVEC tube formation. Figure 4D shows that the hUC-MSCs sheet supernatant groups had obviously better tube nets than the control group. The angiogenesis induced by the hUC-MSCs sheet supernatant group had more junctions and longer distances than the control group.

### Safety of hUC-MSCs sheet

To explore the safety of hUC-MSC sheets, tumorigenicity and cell diffusion experiments after hUC-MSCs sheet transplantation were conducted.

**Fig. 5:**
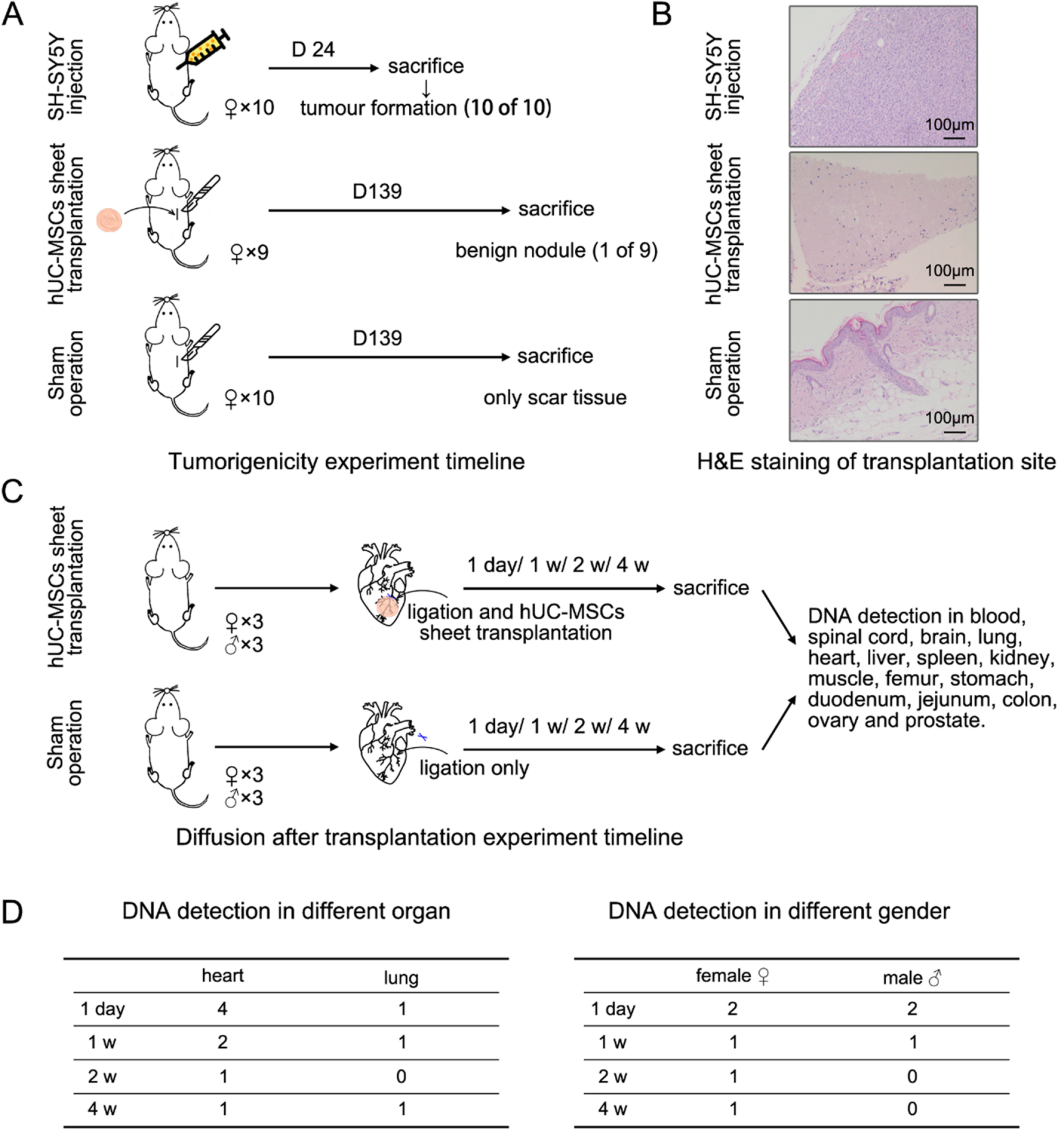
In vivo safety evaluation of hUC-MSCs sheet. **A** Schematic illustrations and timeline of oncogenicity experiment; **B** H&E staining of transplantation site; **C** Schematic illustrations and timeline of diffusion after transplantation experiment; **D** human DNA detection result of diffusion after transplantation experiment

Oncogenicity experiment results showed that all animals in the SH-SY5Y cell injection group had progressive tumor formation. Eight mice had tumors reaching a size of 1,500 mm^3^ at 24 d. The mice were killed in accordance with experimental ethics and tumor was removed for H&E staining analysis (Fig. 5B). For the hUC-MSCs sheet transplantation group, one mouse died after surgery from anaesthesia. Five mice exhibited nodules at 6 d, and these nodules faded away gradually. Finally, at 139 d, only one mouse had a 2.01 mm × 1.73 mm nodule. The histology showed that it was a benign connective tissue nodule without abnormality, as shown by the H&E results in Fig. 5B. All animals in the sham operation group only had scar tissue during the healing stage, which disappeared by 139 d.

All animals in the cell diffusion experiment survived after hUC-MSCs sheet transplantation. No human DNA was detected in the sham operation group. After transplanting the hUC-MSC sheets, human DNA was detected only in the heart and lung at each time point, as shown in Fig. 5D. With prolonged time, the number of animals with human DNA positivity in the heart decreased.

### Efficacy of hUC-MSCs sheet on MI

**Fig. 6:**
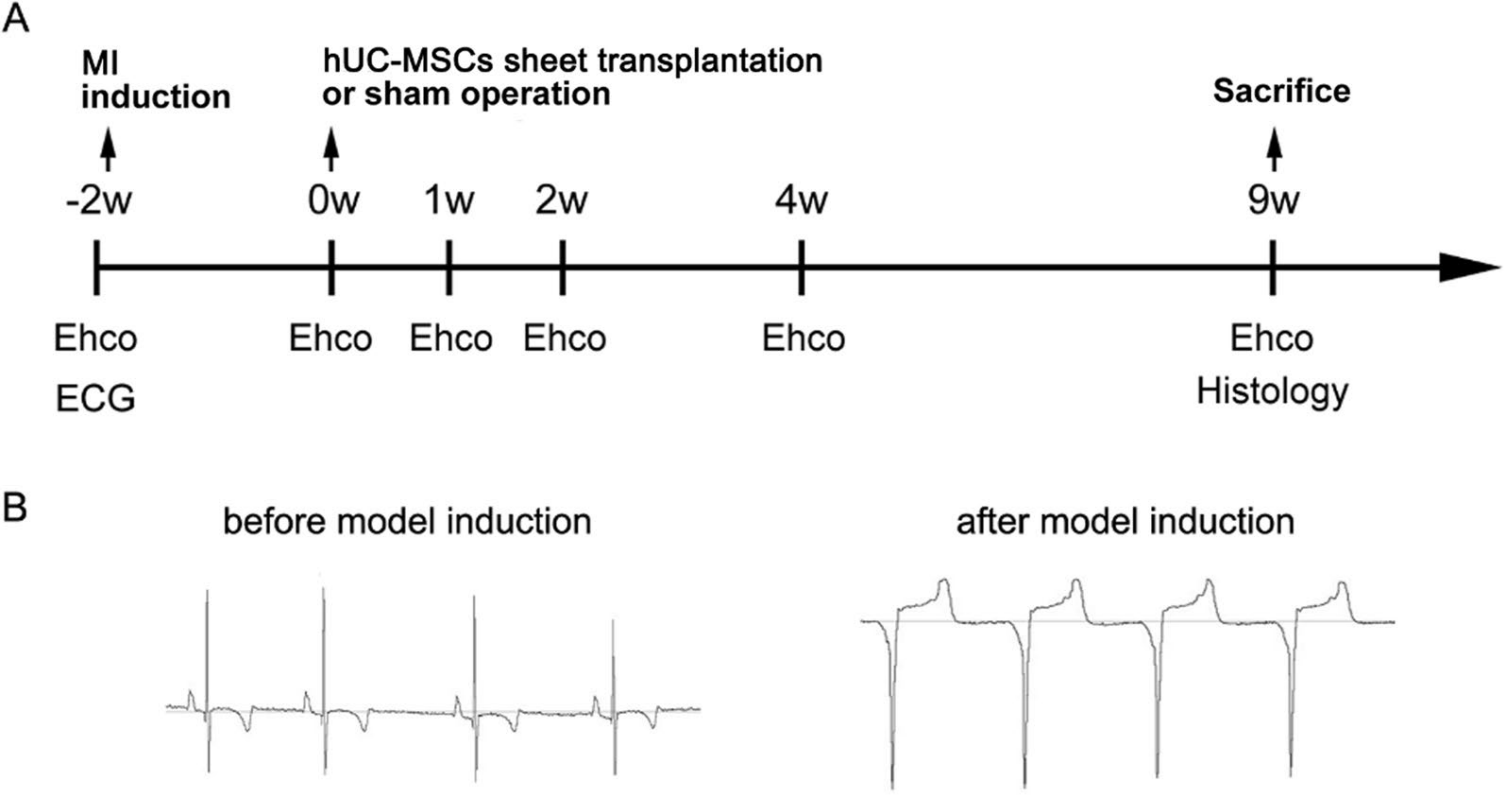
**A** Study protocol of the in vivo availability experiment and the evaluation of cardiac function and histological analysis; **B** Electrocardiogram monitoring of porcine MI model showed ST segment elevation

A flowchart of the animal experiment is shown in Fig. 6A. No animal deaths occurred during the experiment period. The porcine MI model could be reflected through the ECG signal, as shown in Fig. 6B. Animals in both the experimental group and the MI group had a normal ECG before model induction. After model induction, the ST segments were elevated, the T waves had high amplitudes, and deep Q waves occurred in both groups, which demonstrated that myocardial ischemia occurred.

**Fig. 7:**
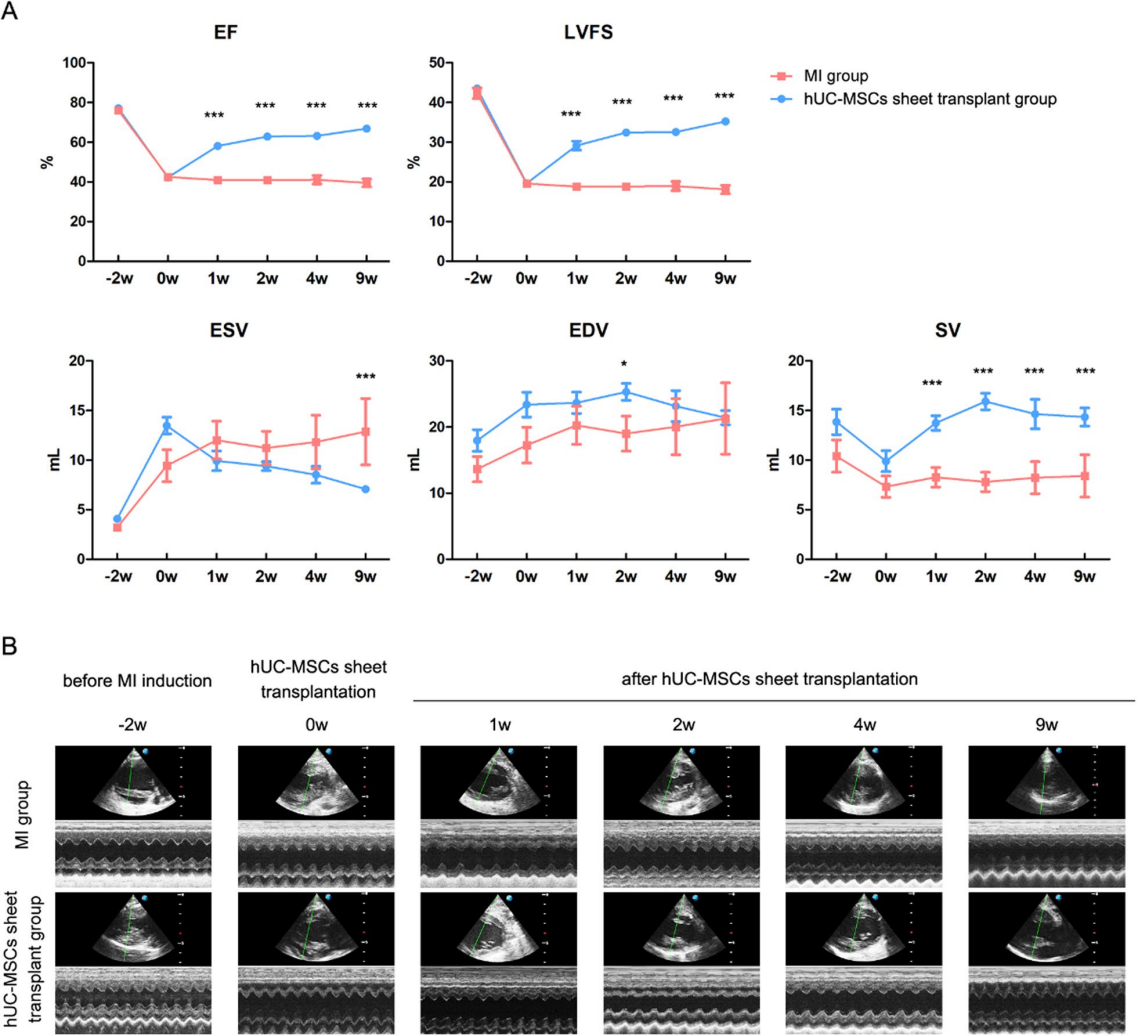
Echocardiographic evaluation of the in vivo availability experiment. **A** Ejection fraction (EF), stroke volume (SV), fraction shortening (FS), end systolic volume (ESV) and end diastolic volume (EDV) of animals in each group; **B** Representative traces of left ventricle short-axis M-mode echocardiograms. *p < 0.05; ***p < 0.001, two groups compared at the same time point

The echocardiography results are shown in Fig. 7. The EF value of the animals decreased from 76.13 ± 1.31% to 42.52 ± 0.65% in the MI group and 77.15 ± 0.43% to 42.25 ± 1.23% in the hUC-MSCs sheet transplantation group due to induced MI (Fig. 7). EF significantly increased to 66.91 ± 1.10% in the hUC-MSC sheet-transplanted groups 9 weeks after surgery. However, the EF value of the MI group animals maintained a flat trend and reached 39.55 ± 1.97% at 9 w. FS had a similar trend for the EF value. ESV and EDV are two other important values to indicate the LV volume condition. The ESV value of the hUC-MSC sheet-transplanted groups was significantly smaller and decreased 0 w after surgery compared with that of the MI group. The EDV value of the hUC-MSC sheet-transplanted groups was larger than that of the MI group. These data indicated that hUC-MSCs sheet transplantation improved left ventricle pumping ability. The SV value of the hUC-MSCs sheet transplantation group was also significantly higher than that of the MI group.

**Fig. 8:**
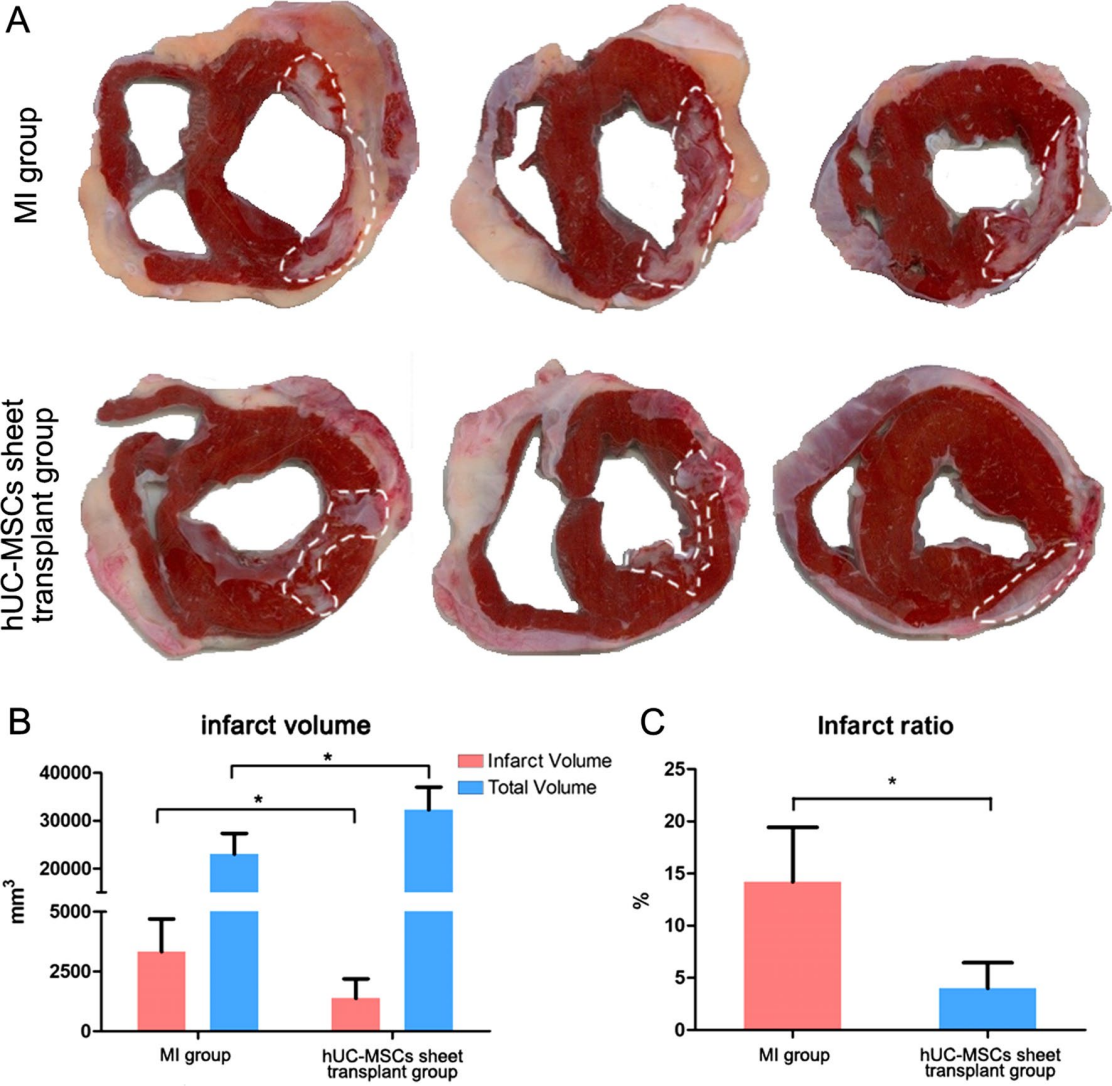
Infarct evaluation of in vivo availability experiment. **A** Macro morphology of heart section in TTC staining, and the orange rectangles indicated the infarct tissue; **B** Infarct volume analysis; **C** Infarct ratio analysis. *p < 0.05

**Fig. 9:**
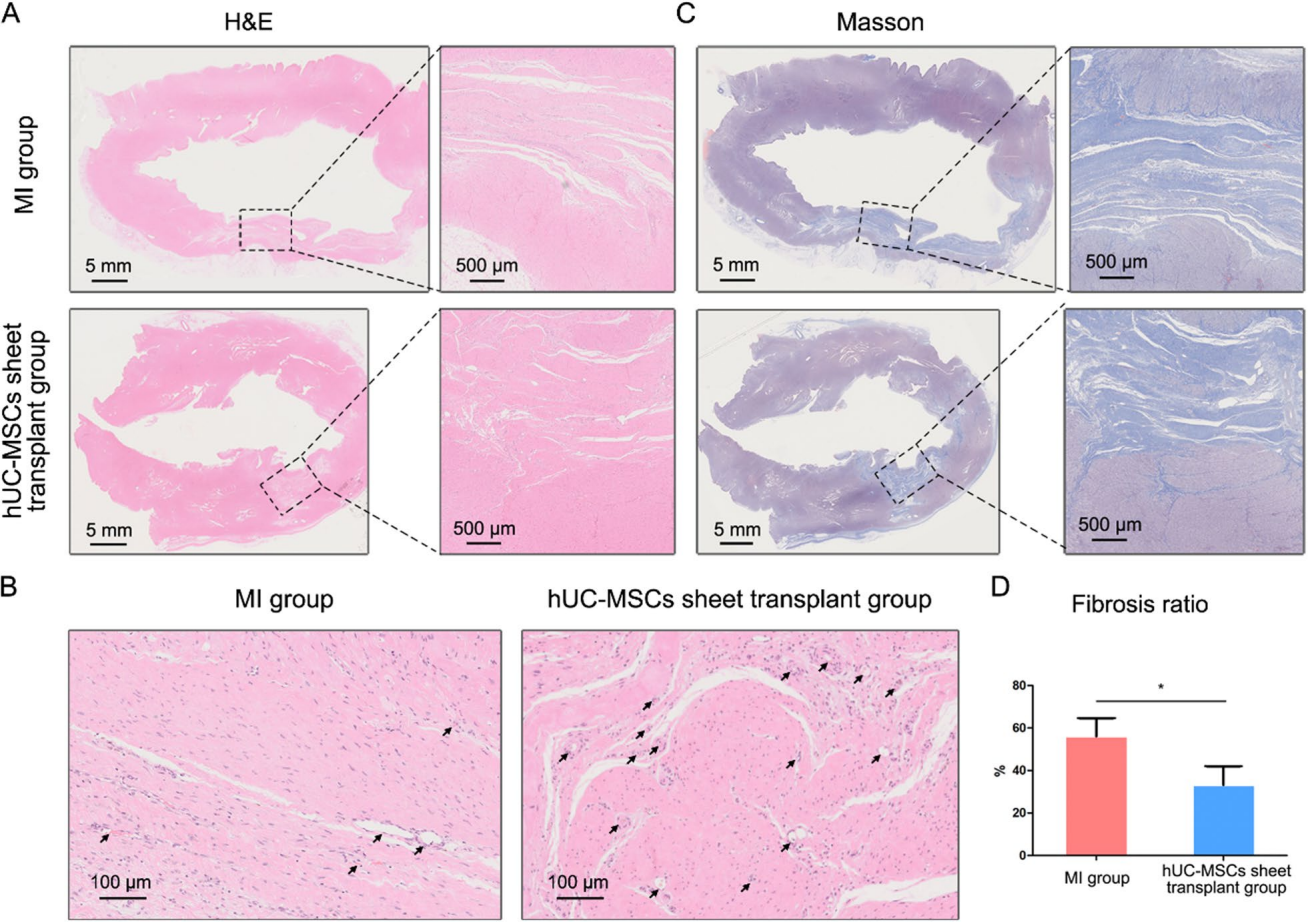
Histology result and analysis of in vivo availability experiment in 9 weeks. **A** H&E staining results of left ventricle in hUC-MSCs sheet transplant group and MI group; **B** H&E staining results in high magnification, and black arrows indicated blood vessels; **C** Masson staining results of left ventricle in hUC-MSCs sheet transplant group and MI group; **D** The fibrosis ratio of infarction location in 9 weeks based on masson staining results

Heart tissues excised nine weeks after transplantation were assessed by histology. The results showed that hUC-MSCs sheet transplantation decreased left ventricle fibrosis. TTC staining provided an intuitive view of the result, and there was a larger grey area (infarct tissue) in the LV myocardium of the MI group than in the hUC-MSCs sheet transplantation group (Fig. 8). Compared to the MI group, animals in the hUC-MSCs sheet transplantation group had a larger LV volume and smaller infarct tissue (Fig. 8); that is, the infarct ratio of the MI group was significantly higher than that of the hUC-MSCs sheet transplantation group (14.2 ± 4.53% vs. 4.00 ± 2.00%, *p* < 0.05, Fig. 8). H&E staining results demonstrated that the myocardium in the hUC-MSCs sheet transplantation group was thicker than the MI group (Fig. 9A), and more blood vessels could be found in the hUC-MSCs sheet transplantation group (Fig. 9B). Masson staining results also revealed that the hUC-MSCs sheet transplantation group had weaker collagen than the MI group, which indicated less fibrosis formation (Fig. 9C). Based on masson staining result, we calculated the fibrosis ratio of the infarction area in both hUC-MSCs sheet transplantation and MI groups (Fig. 9D), which confirmed that the hUC-MSCs sheet transplantation group had smaller fibrosis ratio (33.22 ± 7.11% vs. 56.37 ± 6.94%).

## Discussion

The main strategy of MI therapy is rescuing damaged myocardial cells by restoring blood vessels and downregulating inflammation and fibrosis. hUC-MSCs have potential as a safe and effective way to treat MI, not only because of their paracrine mechanism but also because of their well-established expansion capacity and ethical acceptability. Cell sheet technology makes it possible for hUC-MSCs to perform functions continuously, focused precisely on the surface of the infarction area.

The clinical application of MSCs sheet is the goal of scientific research. Towards this goal, we established cell bank and fabricated hUC-MSC sheets in compliance with Good Manufacturing Practice (GMP). We also established a complete quality control system to assure the microbiological quality and quality consistency (data not shown). In this study, the hUC-MSC sheets fabricated based on the cell bank in a consistent, standardized way. We mainly detected the functional properties of the hUC-MSCs sheet such as cytokines secretion, immunoregulation, and stimulation of angiogenesis in vitro, which are also the widely recognized mechanism of both hUC-MSCs and the hUC-MSCs sheet. In addition, oncogenicity and cell diffusion after hUC-MSCs sheet transplantation were assessed for the first time to demonstrate the safety of the hUC-MSCs sheet. The hUC-MSC sheets transplanted into the heart infarction area porcine MI model showed that they significantly improved cardiac function and reduced the infarct area of the LV. MSC sheets, showing the same mechanics as MSCs with two improvements: augmentation of cytokine production and ECM retention.

Emerging research suggests that a paracrine effect is the main mechanism underlying the ability of MSCs to induce cardiac repair rather than differentiating into cardiac cell lineages [29–31]. In this study, the transplantation of hUC-MSCs sheet into porcine MI model was beneficial to improve LV function, which can be due to the cytokine secretion effect. Compared to MSC suspensions, MSC sheets experience structural and morphological transitions during detachment from thermo-responsive culture dishes, which increases their cell–cell and cell–matrix interactions. This structural property of MSC sheets enhances their MSC cytokine secretory capacity [32]. We detected that hUC-MSCs sheet had a higher cytokines secretion than normal cultured hUC-MSCs (see Supplementary). Normal cultured hUC-MSC hardly secret VEGF, while the supernatant of hUC-MSCs sheet has around 4000–7000 ng/ml VEGF in the supernatant. In this study, we also detected IL-6, IL-8, and HGF, which are the representative of the various cytokines secreted by MSCs (Additional file 1: Figure S1). Cytokines work on the MI by (1) anti-inflammation, (2) reduction of fibrosis, and (3) stimulation of angiogenesis. Adverse LV remodelling of the MI is related to a chronic inflammatory response [33].

A multicentre clinical trial showed that TNF and its receptors (TNFR1 and TNFR2) were associated with increased mortality among advanced HF patients [34]. The present in vitro theory is that MSCs can suppress the proliferation of dendritic cells and the activation of T cells and natural killer cells to diminish TNF-α secretion and increase IL-10 secretion [35]. MSCs cause TH1 cells to decrease secretion of IFN-γ, TH2 cells to increase the secretion of IL-4 and NK cells to decrease the secretion of IFN-γ [35]. Our in vitro immunoregulation results are consistent with these findings. Immunoreaction induced by the MI is usually caused by the fibrosis in the infarct area and it eventually leads to adverse remodelling [33]. The in vivo results in this study showed that hUC-MSC sheets can effectively decrease the infarct ratio of a porcine MI model. Masson staining results also demonstrated weaker fibrosis conditions in the hUC-MSC-transplanted groups. These results are in accord with previous research, although different MSC sources were used [13, 14].

Ischemia and necrotic cardiomyocytes are the direct consequences of MI; hence, angiogenesis induction by MSCs is an effective mechanism of treatment. Angiogenesis involves the coordination of a very large number of factors secreted by MSCs. [36]. MSC sheets can secrete extra VEGF to stimulate angiogenesis [32, 37]. In addition, VEGF-independent mechanisms promote angiogenesis, such as CXCL1, CXCL5, CXCL6, IL8, and HGF [38]. In this study, conditioned medium of hUC-MSC sheets had a strong promoting effect on HUVEC angiogenesis manifested in the form of more junctions and longer tube lengths than the MI group due to cytokines secretion of the hUC-MSCs sheet. The angiogenesis results also provide a reasonable explanation for the decrease in the fibrosis ratio in vivo. In this study, we observed more vessels in the ischemic region of mini-pigs’ LV in hUC-MSCs sheet transplanted group than MI group. In another ongoing study, the same mini-pig MI model was used to evaluate the validity of hUC-MSCs sheet on MI for long term. Available results showed that more positive α-SMA staining could be observed in the ischemic region in the hUC-MSCs sheet transplanted group than MI group 4 weeks after hUC-MSCs sheet transplantation (Additional file 1: Figure S2), which confirmed the hUC-MSCs sheet transplantation has a positive effect on angiogenesis (see Additional file 1). Long-term observation is still ongoing.

As mentioned above, ECM retention is another advantage of cell sheet technology, which allows MSC sheets to work at a higher density and have better retention properties than MSC suspensions. Figure 3 shows that abundant ECM existed in the SEM observations and histological staining. The retention of ECM in MSC sheet fabrication can prevent anoikis and the apoptosis that occurs due to cell detachment from the ECM [39]. In that way, MSC sheets have improved curative effects in HF [16, 40]. Moreover, our previous study also showed that the ECM of hUC-MSC sheets provides a scaffold to recruit endogenous cells to form new vascular structures and promote angiogenesis [25].

MSCs are recognized as safe in preclinical [5] and clinical [4, 41, 42] use for heart disease. However, there are still occasional reports about cellular abnormalities, such as tumorigenesis [43, 44]. It is worth highlighting that MSC sheets are not only an accumulation of cells but also an amplification of function. Therefore, safety evaluation targeting hUC-MSC sheets is necessary. The results showed that hUC-MSC sheets did not become tumors after transplantation, which demonstrated that the accumulation of hUC-MSCs did not change the characteristics of the MSCs or promote the differentiation of MSCs. To identify whether the hUC-MSCs sheet fabrication process will affect the characteristics of hUC-MSCs, the hUC-MSCs sheet was digested into single cells to identify their adherence, differentiation ability, and surface markers. Therefore, hUC-MSC sheets have no risk of tumorigenicity. The results showed that the MSCs in the hUC-MSCs sheet still met the criteria of MSCs. Unlike MSC suspension administration, MSC sheets do not spread through the body through blood flow or leak from the injection site. After transplantation of hUC-MSC sheets onto the surface of the heart tissue, the hUC-MSCs were mainly distributed to the heart and lungs because cardiopulmonary circulation, and no human DNA or fragments of hUC-MSC sheets were detected in other organs or tissues. This result is also evidence for the concentration effect of hUC-MSC sheets. As our previous study has reported, there were no human hUC-MSCs remaining at 4 weeks after hUC-MSCs sheet transplantation in mice MI model [25]. In this study, various cytokines were produced by UC-MSC sheets, and these cytokines contributed to improvement of cardiac function by inhibiting apoptosis of cardiomyocytes and inducing therapeutic angiogenesis of the porcine infarcted heart since the main purpose of this study to evaluate the safety of hUC-MSC sheets in vitro and in vivo, and the number of effectiveness experiments is limited. In-depth studies of the specific mechanism underlying the therapeutic effects of hUC-MSC sheets should be carried out to verify by more porcine MI models in further studies.

There are still many aspects that need to be improved. Firstly, although this moderate benefit corresponds better to the desired results of a clinical study, giving realistic insight into the expected benefit of human cell therapies, the number of large animals is limited in this study. In future experiments, the number of large animals needs to be increased to better observe the therapeutic effect of hUC-MSC sheets. Secondly, a in vivo real-time monitoring method for heart function should be added to future evaluations to provide a whole course tracing, which may provide a clear timeline of the hUC-MSCs sheet working cycle after cell sheet transplantation. Thirdly, there are inter sample differences exist among umbilical cords, which could be seen during the in vitro testing results. We will collect additional samples to expand the hUC-MSC bank and thus develop a stricter standard for usable hUC-MSCs. Moreover, a quality assessment protocol will also be developed and published in the future. Another method to minimize differences between samples is using human-induced pluripotent stem cell-derived mesenchymal stem cells (iPSC-MSCs), which has been a research hotspot in cell therapy for years. Researchers have demonstrated the safety of iPSC-MSCs with no tumor formation and lower showed stronger immune privilege property than BM-MSCs [45, 46]. iPSC-MSCs showed good immunomodulation property and angiogenic effects via paracrine effects. In addition, iPSC-MSCs also secure damaged cardiomyocytes by mitochondrial transfer [47]. Whatever hUC-MSCs or iPSC-MSCs, only by confirming the safety and effectiveness of preclinical studies in large animals should it be moved to clinical trial.

## Conclusion

In this study, an hUC-MSCs sheet was fabricated based on the establishment of a cell bank of hUC-MSCs under strict quality control. In vitro assays showed that the hUC-MSCs sheet secrete more VEGF, HGF, IL-6, and IL-8, which contribute to immunoregulation and angiogenesis acceleration. After implantation in vivo, the hUC-MSCs sheet showed no risk of oncogenicity. hUC-MSC sheets transplantation improved cardiac function, diminished the fibrosis ratio, and attenuated LV remodelling in a porcine MI model. Thus, the hUC-MSCs sheet makes it possible to improve the function of a failing heart through a manufactured product with universal quality instead of individualized medical technology.

## Supplementary Information

**Additional file 1. Figure Sup 1.** Comparation of cytokines secretion between hUC-MSCs and the hUC-MSCs sheet. *** means *p* < 0.001. **Figure Sup 2.** α-SMA staining (indicated by black arrows) of ischemic region of mini-pigs’ LV in hUC-MSCs sheet transplant group and MI model group.

## Data Availability

Not applicable.

## Abbreviations

AD-MSC: Adipose mesenchymal stem cell
BM-MSC: Bone marrow mesenchymal stem cell
ECG: Electrocardiography
ECM: Extracellular matrix
EDV: End diastolic volume
EF: Ejection fraction
ESV: End systolic volume
FS: Fraction shortening
GMP: Good Manufacturing Practice
HF: Heart failure
HGF: Hepatocyte growth factor
hUC-MSC: Human umbilical cord mesenchymal stem cell
H&E: Haematoxylin and eosin
iPSC-MSCs: Induced pluripotent stem cell-derived mesenchymal stem cells
LAD: Left coronary artery anterior descending branch
LV: Left ventricular
LVEF: Left ventricular ejection fraction
MI: Myocardial infarction
MSC: Mesenchymal stem cell
PBMC: Peripheral blood mononuclear cell
SV: Stroke volume
TTC: 2,3,5-Triphenyltetrazolium chloride
UC-MSC: Umbilical cord mesenchymal stem cell
