# Supplementary material for: Preclinical study of human umbilical cord mesenchymal stem cell sheets for the recovery of ischemic heart tissue
Source: Stem Cell Res Ther. 2022 Jun 11;13:252. doi: 10.1186/s13287-022-02919-8 (PMC9188245; doi:10.1186/s13287-022-02919-8)
Supplement: Supplementary file 1 — Additional file 1. Figure Sup 1. Comparation of cytokines secretion between hUC-MSCs and the hUC-MSCs sheet. *** means p < 0.001. Figure Sup 2. α-SMA staining (indicated by black arrows) of ischemic region of mini-pigs’ LV in hUC-MSCs sheet transplant group and MI model group. [file 13287_2022_2919_MOESM1_ESM.docx]

Preclinical study of human umbilical cord mesenchymal stem cell sheets for the recovery of ischemic heart tissue

Shuang Gao ^1^, Yongqiang Jin ^2^, Jianlin Ma ^1^, Juan Wang ^1^, Jing Wang ^1^, Zehua Shao ^3^, Taibing Fan ^4^_,_ Mingkui Zhang ^2^, Dehua Chang ^5, *^

^1^ BOE Regenerative Medicine Technology Co., Ltd., No. 9 JiuXianQiao North Road, Beijing 100015, China.

^2^ Heart Center, First Hospital of Tsinghua University, No. 6 JiuXianQiao 1st Road, Beijing 10016, China.

^3^ Heart Center of Henan Provincial People's Hospital, Zhengzhou University People's Hospital, No. 7 Weiwu Road, Zhengzhou, 450003, China.

^4^ Children Heart Center, Fuwai Central China Cardiovascular Hospital, No. 1 Fuwai Road, Zhengzhou 450018, China.

^5^ Department of Cell Therapy in Regenerative Medicine, The University of Tokyo Hospital, 7-3-1 Honggo, Bunkyo-ku, Tokyo 113-8655, Japan.

^*^ Correspondence to Dr. Dehua Chang, Department of Cell Therapy in Regenerative Medicine, The University of Tokyo Hospital, 7-3-1 Honggo, Bunkyo-ku, Tokyo 113-8655, Japan. jot.sur@mail.u-tokyo.ac.jp; dehua_chang@yahoo.com.

**Comparation of cytokines secretion between hUC-MSCs and the hUC-MSCs sheet**

hUC-MSCs at the working cell bank were thawed and cultured at T-75 culture flask by

culture medium to around 70% confluence. Then, the culture medium was sucked out and the fresh culture medium were added for 24 h incubation at 37 °C, 5% (v/v) CO_2_, and 95% humidity. The medium was collected for IL-6 (R&D, Minneapolis, USA), IL-8 (R&D), VEGF (R&D), and HGF (Invitrogen, Camarillo, USA) detection by enzyme-linked immunosorbent assay. Cytokines secretion from hUC-MSCs sheet are same with the results in the main manuscript. The results were compared with cytokines secreted by hUC-MSCs sheets as follows (Figure Sup.1).


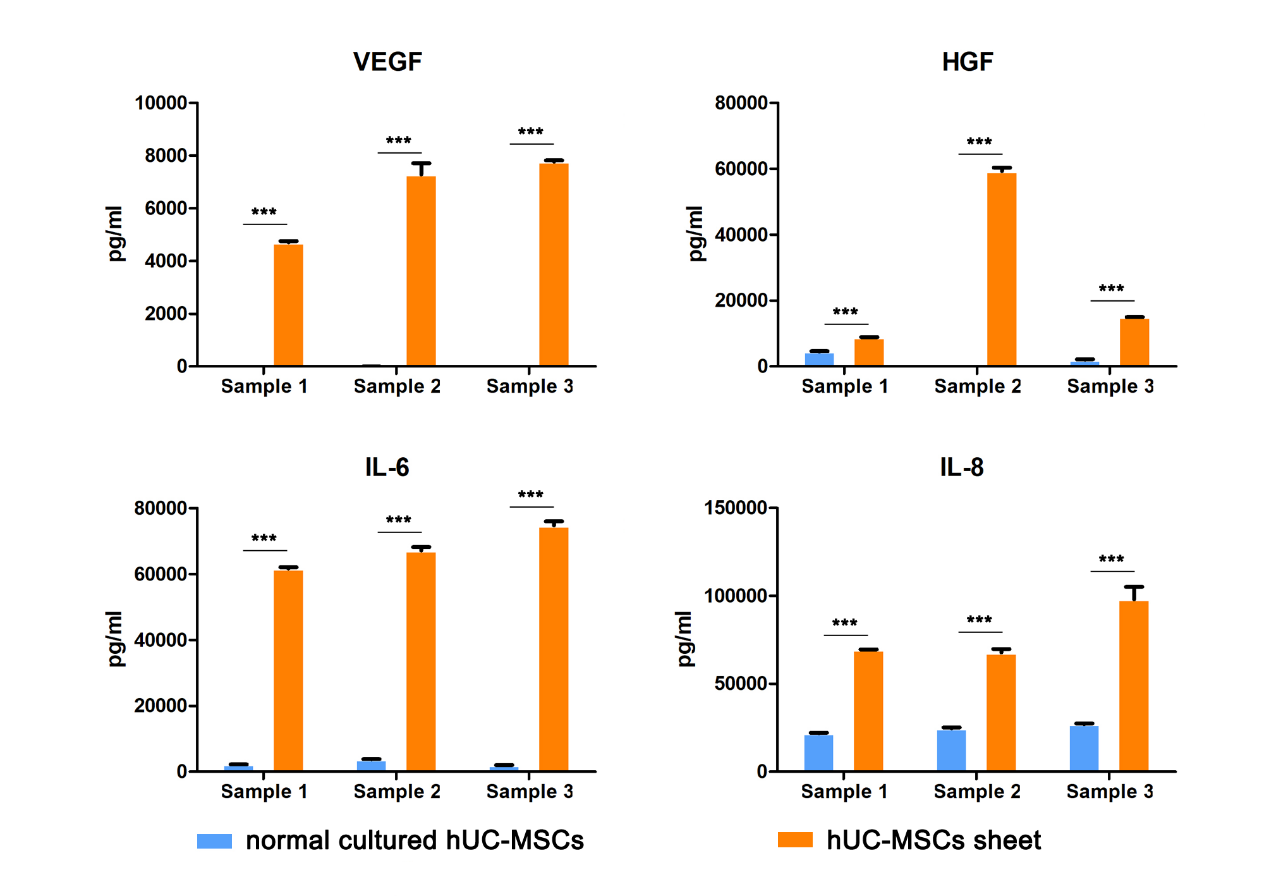


Figure Sup.1. Comparation of cytokines secretion between hUC-MSCs and the hUC-MSCs sheet. *** means p<0.001.

**α-SMA staining of ischemic region of mini-pigs’ LV in hUC-MSCs sheet transplant group and MI model group**

This result is from an ongoing study. In this study, a hUC-MSCs sheet was transplanted onto the LV infarcted area of MI mini-pigs. The hUC-MSCs sheet fabrication, MI model making and hUC-MSCs sheet transplantation are same as the main manuscript. The main purpose of this study was to evaluate the validity and safety of hUC-MSCs sheet on MI mini-pigs for 25 weeks. There were more check points than the validity experiment in the main manuscript. 4 weeks after hUC-MSCs sheet transplantation, animals in this point were euthanized, and the hearts were removed and fixed with 10% formalin for histological analysis. The infarct tissue of the heart was stained with anti-α smooth muscle actin (Servicebio, Wuhan, China). Results showed that more positive α-SMA staining could be observed in the ischemic region of mini-pigs’ LV of the hUC-MSCs sheet transplanted group than MI group (Figure Sup.2), which confirmed the hUC-MSCs sheet transplantation has a positive effect on angiogenesis. Longer observation is still ongoing.


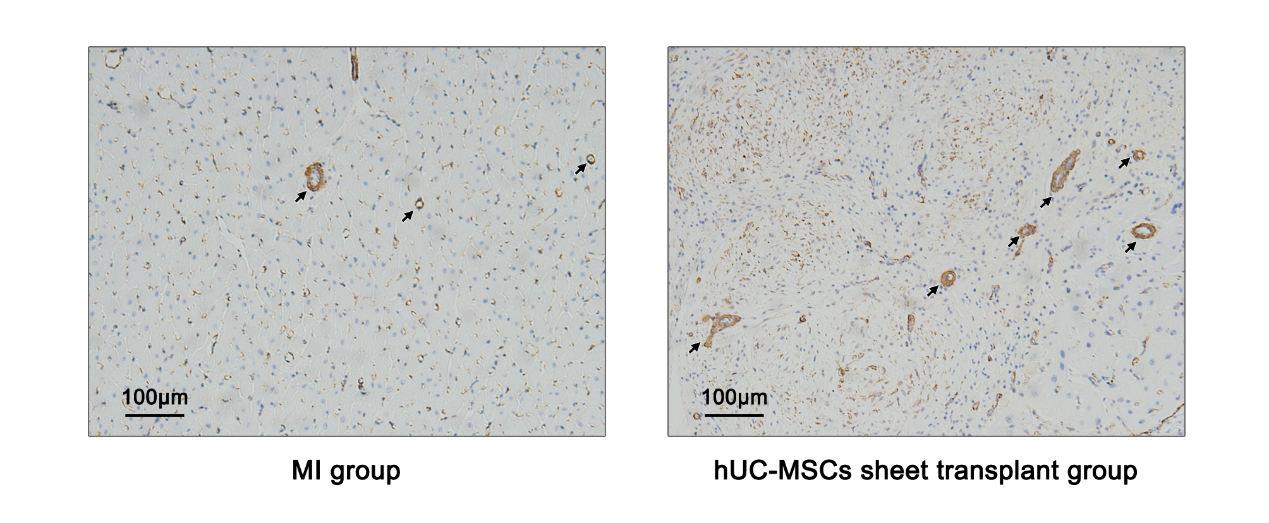


Figure Sup.2. α-SMA staining (indicated by black arrows) of ischemic region of mini-pigs’ LV in hUC-MSCs sheet transplant group and MI model group
